# MIC: Microwave Imaging Curtain for Dynamic and Automatic Detection of Weapons and Explosive Belts

**DOI:** 10.3390/s23239531

**Published:** 2023-11-30

**Authors:** Rémi Baqué, Luc Vignaud, Valentine Wasik, Nicolas Castet, Reinhold Herschel, Harun Cetinkaya, Thomas Brandes

**Affiliations:** 1 ONERA/DEMR, F-13661 Salon de Provence, France; nicolas.castet@onera.fr; 2 ONERA/DEMR, Université de Paris-Saclay F-91123 Palaiseau, France; luc.vignaud@onera.fr; 3 ONERA/DEMR, Université de Toulouse, F-31055 Toulouse, France; valentine.wasik@onera.fr; 4 Fraunhofer FHR, D-53343 Wachtberg, Germany; reinhold.herschel@fhr.fraunhofer.de; 5 Institute of Integrated Systems, Ruhr-Universität Bochum, D-44780 Bochum, Germany; harun.cetinkaya@ruhr-uni-bochum.de; 6 Fraunhofer SCAI, D-53757 Sankt Augustin, Germany; thomas.brandes@scai.fraunhofer.de

**Keywords:** ultra-wide band radar, artificial intelligence, 3D radar, MIMO radar, counter-terrorism

## Abstract

DEXTER (detection of explosives and firearms to counter terrorism) is a project funded by NATO’s Science for Peace and Security (SPS) program with the goal of developing an integrated system capable of remotely and accurately detecting explosives and firearms in public places without impeding the flow of pedestrians. While body scanner systems in secure areas of public places are becoming more and more efficient, the attack at Brussels airport on 22 March 2016, upstream of these systems, in the middle of the crowd of passengers, demonstrated the lack of discreet and real-time security against threats of mass terrorism. The NATO-SPS international and multi-year DEXTER project aims to provide new technical and strategic solutions to fill this gap. This project is based on multi-sensor coordination and fusion, from hyperspectral remote laser to smart glasses, artificial algorithms, and suspect identification and tracking. One of these sensors is dedicated to threat detection (large weapon or explosive belt) using the clothing of pedestrians by means of an active microwave component. This project is referred to as MIC (Microwave Imaging Curtain), also supported by the French SGDSN (General Secretariat of Defense and National Security), and utilizes a radar system capable of generating 3D images in real-time to address non-checkpoint detection of explosives and firearms. The project, led by ONERA (France), is based on a radar imaging system developed by the Fraunhofer FHR institute, using a MIMO architecture with an Ultra-Wide Band waveform. Although high-resolution 3D microwave imaging is already being used in expensive body scanners to detect firearms concealed under clothing, MIC’s innovative approach lies in utilizing a high-resolution 3D imaging device that can detect larger dangerous objects carried by moving individuals at a longer range, in addition to providing discrete detection in pedestrian flow. Automatic detection and classification of these dangerous objects is carried out on 3D radar images using a deep-learning network. This paper will outline the project’s objectives and constraints, as well as the design, architecture, and performance of the final system. Additionally, it will present real-time imaging results obtained during a live demonstration in a relevant environment.

## 1. Introduction

Securing passengers and revealing risks have become a growing challenge in the face of increasing populations and varying threats. There is, therefore, an increasing demand for detection systems that can reveal risks to the public.

A great deal of progress has indeed been made in developing body scanners and material detectors for security checkpoints in airports and entrances to public places. These devices are able to detect small hidden objects due to their very high resolution but require the cooperation of the suspect and lengthy scanning, and they are not discreet. These disadvantages slow down traffic, are sometimes intrusive, and generate a vulnerable queue ahead of the control.

The Brussels airport terrorist attack on 22 March 2016 took advantage of these shortcomings by massively attacking the public gathered ahead of the checkpoints.

Regarding this, there is a need for discreet devices that can reveal the risk while individuals walk in a continuous flow.

Although this need is evident, numerous research studies, both in the microwave [1,2,3,4] and millimeter-wave [5,6] regions, have focused on stationary scenarios where an individual must remain stationary and be illuminated for several seconds. In the coming years, due to the growing number of passengers, such solutions will not be sufficient to cope with the anticipated growth within a reasonable timeframe.

**Figure 1:**
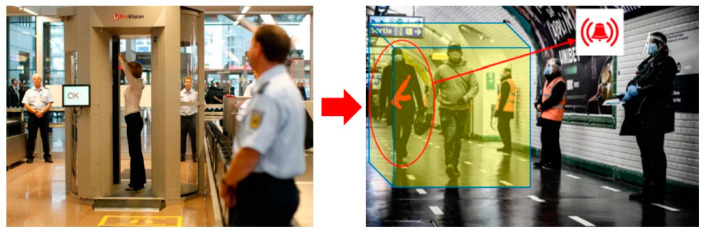
Objective to evolve from short-range detection at checkpoints to automatic discreet detection in the passenger flow.

The objective of the MIC (Microwave Imaging Curtain) is to serve as a proof-of-concept prototype for the automatic standoff detection of firearms and explosive belts hidden beneath the clothing of individuals walking in a continuous flow. Microwave signals possess the capability to penetrate non-metallic materials, and millimeter wavelength sensors are already in use for passenger inspections prior to aircraft boarding [4]. However, the individual under inspection must remain stationary at the center of the scanner, being illuminated for several seconds (Figure 1 left). These detection systems have numerous drawbacks, including their lack of discretion and the creation of a crowd of vulnerable people ahead of the checkpoint.

MIC seeks to complement this detection principle by automating the inspection of a continuous flow of individuals in a much larger search volume and on a much smaller timescale. This challenge could be met by considering larger objects to be detected than in aircraft boarding scenarios (e.g., automatic rifles instead of small ceramic knives). The scientific and technical challenges relate to real-time and large observable volume performances, with the possibility of reducing the resolution constraint necessary to detect and identify the targeted threats (Figure 1 right).

The MIC project aims to design, develop, and test a radar-based imaging device in a representative environment that addresses non-checkpoint firearms detection issues, which are increasingly challenging for operators of mass transportation systems and organizers of large public events. In accordance with current regulations regarding the impact of radiation on human health and privacy protection, the project integrates off-the-shelf high-performance microwave modules and develops specific signal processing algorithms to reconstruct 3D images of objects carried by moving individuals in the field of view of transmitting modules and scattered-wave-receiving modules. Post-processing of such images will perform automatic detection of dangerous objects.

## 2. Description of the Imaging System Hardware

### 2.1. System Modular Structure

**Figure 2:**
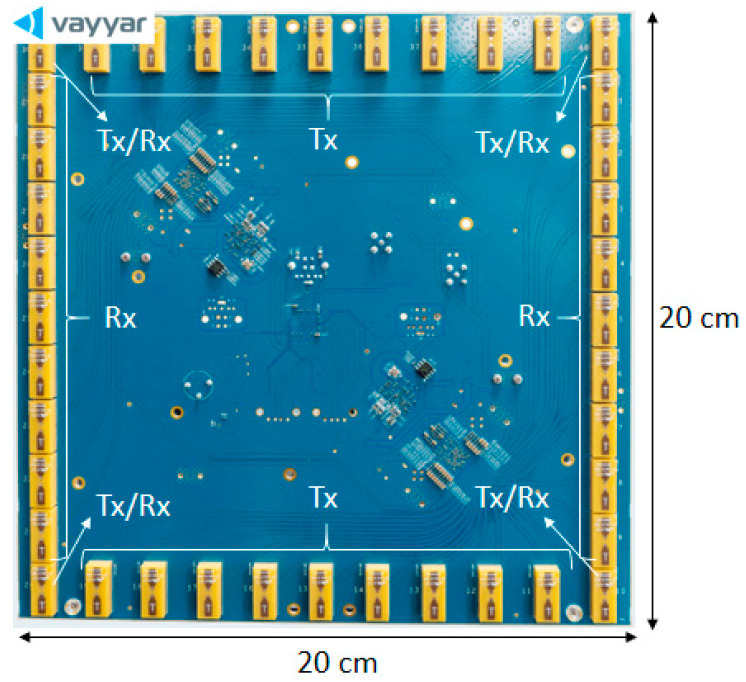
COTS module with transmitter and receiver antenna array.

The system is based on a modular structure. Each module (being a COTS, commercial off-the-shelf device, produced by the Vayyar company [7]) in Figure 2 consists of 20 transmitter (Tx) and 20 receiver (Rx) elements that are distributed along a quadratic perimeter. The array size is 20 cm in both x-y-axes. The module operates at 8.5 GHz central frequency with 4 GHz bandwidth (3.53 cm central wavelength). The transmitted radar waveform is a classic Step-Frequency Continuous Wave (SFCW). The 3 dB antenna aperture is about 100°.

**Figure 3:**
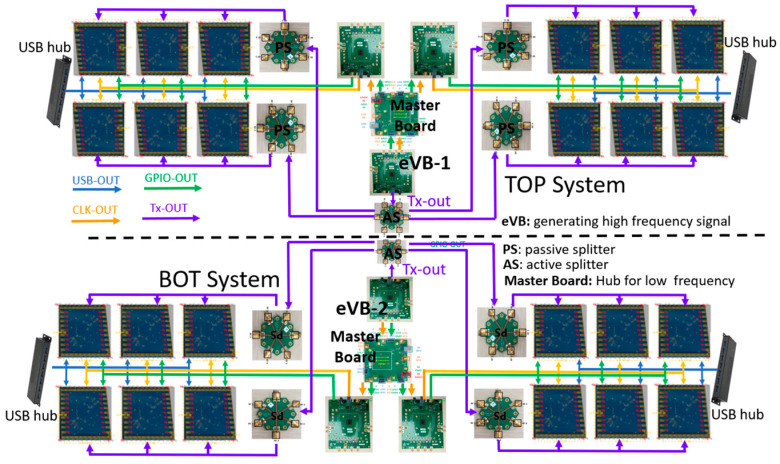
Tree structure assembly of the 24 COTS transmit and receive modules in two sub-systems (TOP and BOT).

The complete imager is composed of two independent sub-systems: a top one (TOP) and a bottom one (BOT), mounted in a stack on the vertical axis. Each sub-system is built in a tree structure that combines 12 MIMO (Multiple Input Multiple Output) modules (Figure 3). This MIMO technique enables the creation of a large and high-resolution virtual antenna [8]. Each sub-system operates completely coherently since, instead of using different Tx/Rx modules for each antenna (which could introduce a slight difference in the transmitted signals), the RF signal for each transmitter element is generated by a single source placed on a central Master Board. The generated signal is then distributed through passive and active signal splitters to every module (Figure 3).

It should be noted that four of the antennas are operational as both transmitter and receiver elements on the module (Figure 2). Thus, despite having 40 antennas, there are 22 Tx and 22 Rx antennas on each module. The whole system, including the TOP and BOT sub-systems, consists of 24 modules with 528 transmitter and 528 receiver antennas. In order to reduce the total measurement time, the transmitter antennas that are located on the external modules are turned off. Even though this approach generates a reduced resolution in the x-axis, the radar system is still able to provide a horizontal resolution of about a centimeter. A total of 352 transmitter antennas are then in use while all receiver antennas are employed in both sub-systems. Each sub-system works independently, meaning that the Rx antennas on one sub-system only receive signals from the Tx antennas of this sub-system. This approach reduces the total number of antenna pairs to 92,928, half of which come from the TOP sub-system and the rest from the BOT sub-system. The conceived design then results in reducing the complexity of the final imaging system and the computational cost of the radar imaging. Moreover, this strategy reduces the total transmitted energy but it is still sufficient with respect to the expected range.

The system hardware parameters are summarized in Table 1.

### 2.2. The MIC System Configuration

The flexible tree structure allows for many antenna array configurations. After studies and simulations, as a compromise between cross-range resolutions, system discretion, and control of the trajectory of the persons to be imaged, a door configuration was selected with a width of 87 cm between each panel, which is typical of a standard office door. Passengers walk through the MIC sensor, one by one. Wider passages could be useful, particularly for improving the passenger flow, but could lead to artefacts in radar images and loss of detection performance. This point is discussed in Section 4.

**Figure 4:**
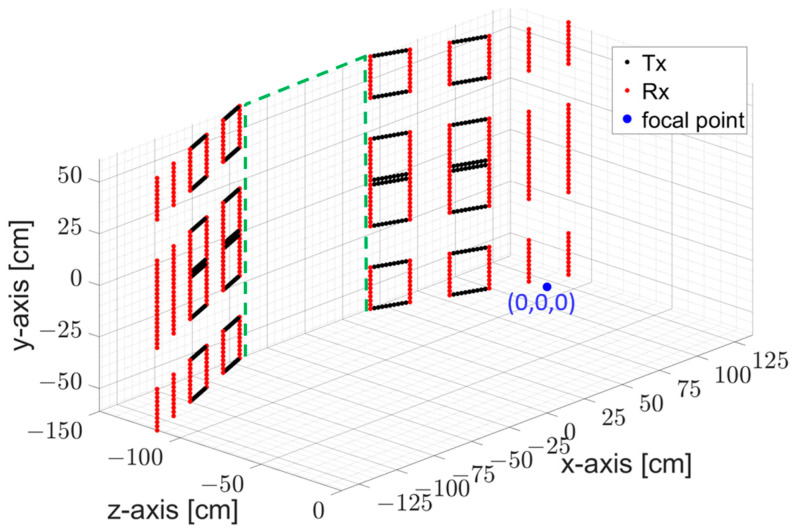
MIC door configuration based on 24 COTS transmit and receive modules organized in two sub-systems (TOP and BOT). The 87 cm-wide door is indicated as a green dotted line.

A total of 24 panel sub-systems are assembled one on top of the other to image a volume from 0.8 to 2 m above ground on the y-axis, and from −0.5 to 0.5 m on the x-axis (Figure 4). The 81 frequency points and 4 GHz bandwidth indicated in Table 1 allow for non-ambiguous image formation at up to 3 m depth on the z-axis. Panels are oriented to focus energy from 0.5 to 2.5 m from the door threshold on the z-axis.

### 2.3. The MIC System Connection to Workstations

**Figure 5:**
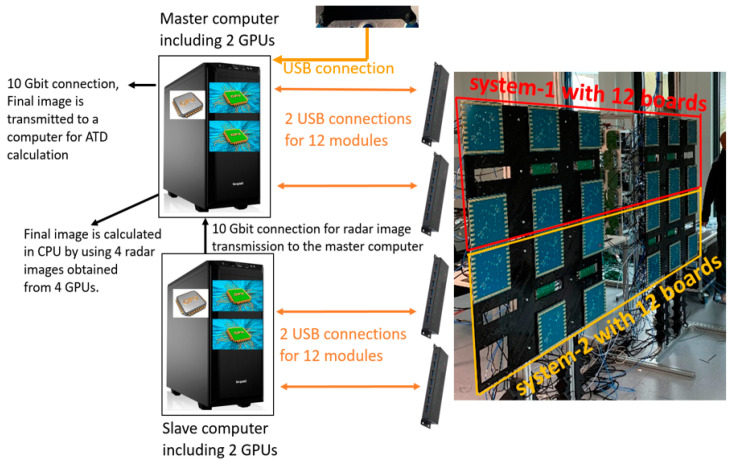
Overall system with 2 workstations including 4 GPUs.

The imaging system with connections to the corresponding workstations is shown in Figure 5. The first workstation is the master, connected to the TOP sub-system, and the second is the slave, connected to the BOT sub-system. Each sub-system has two USB connections for radar data recording. Both workstations are equipped with two GPUs for real-time image processing. Therefore, a total of four GPUs process four radar images. The slave workstation sends the results to the master workstation to centralize all the data. Finally, the master workstation uses its CPU to compute the final image from the four radar images and sends it via a 10 Gbit connection to a computer performing the Automated Target Detection (ATD). 

Once the measurements and processing are completed, only the final image, a 3D matrix of complex data, is saved.

### 2.4. The Imaging System Algorithm

#### 2.4.1. Imaging Algorithm

The algorithm utilized by the imaging system is a critical aspect of the radar image, as it incorporates numerous signal-processing functions that ultimately reveal the focused targets.

**Figure 6:**
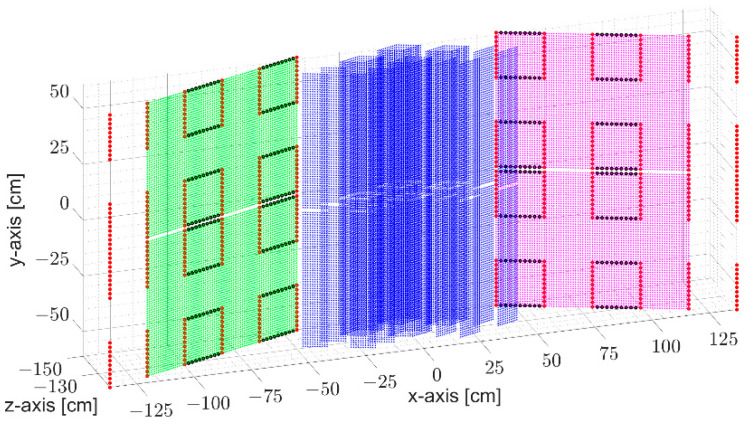
Virtual elements formed from the proposed MIMO array design. Green and magenta-colored virtual elements are formed from transmitter and receiver antennas, both on the negative or positive x-axis. Blue-colored virtual elements are formed from transmitter and receiver antennas, placed at different sides of the x-axis.

Prior to addressing the imaging algorithm, since some of the applied filters in the imaging algorithm are closely related to the position of virtual elements, a brief explanation regarding a virtual array formed from the proposed MIMO system design is necessary. As explained in Section 2.1, the MIMO system comprises 352 transmitters and 528 receivers divided into two sub-systems. For each sub-system, 46,464 signals can then be extracted, which means that each sub-system can be seen as a 46,464-element virtual antenna array. As shown in Figure 6, green and magenta-colored virtual elements are formed from transmitter and receiver antennas, both on the negative or positive x-axis, respectively, and distributed over a 2D plane. Blue-colored virtual elements are formed from transmitter and receiver antennas, placed at different sides of the x-axis, and distributed within the 3D spatial domain. Even though the virtual array provides uniform distribution over the x-y-axes, their co-locations and distributions within the 3D spatial domain result in degraded image quality. The imaging algorithm, therefore, also removes these degrading features.

**Figure 7:**
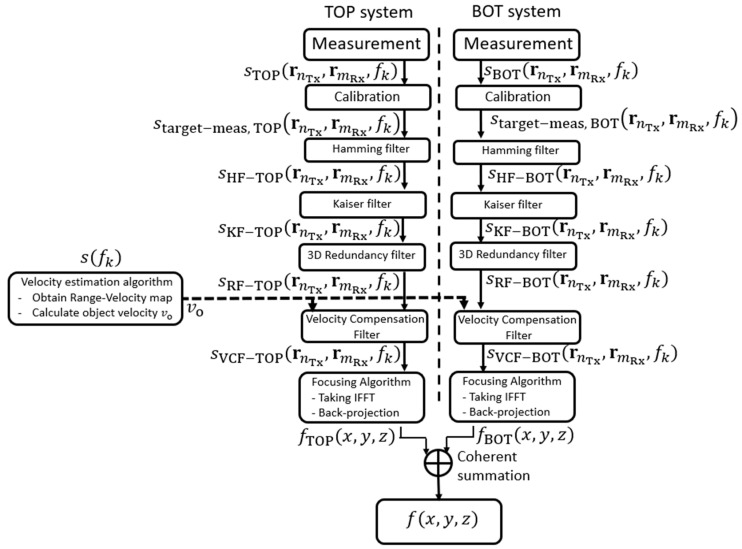
Imaging algorithm with functions.

Figure 7 summarizes the procedures of the imaging system algorithm. Two sub-systems (TOP and BOT) in the radar imaging system illuminate the region of interest with SFCW and save the scattering parameters in complex format within the frequency band for every transmit/receive pair. Calibration coefficients are then applied to the measurement data using the active calibration method. A Hamming filter is applied to the data along the frequency range for every transmit/receive pair to suppress the range side lobes. A Kaiser filter is subsequently applied to the measurement data to suppress the lateral side lobes. Afterwards, a 3D multiplicity filter is employed to obtain more homogenous illumination over the 2D array aperture. A velocity compensation filter is applied to compensate for the phase difference due to the person’s movement. This is followed by inverse fast Fourier transform (IFFT), which is used to obtain the signal in the spatial domain. A back-projection (BP) algorithm is applied to focus the data for a complex 3D image. Finally, the 3D complex radar image in the BOT sub-system is first transferred to the master workstation, and then a coherent summation is performed to obtain the final 3D complex radar image by using two 3D complex radar images from the TOP and BOT sub-systems.

The following is a concise exposition describing the processing and evaluation of scattering parameters from two distinct sub-systems, ultimately yielding a comprehensive 3D complex radar image.

**Figure 8:**
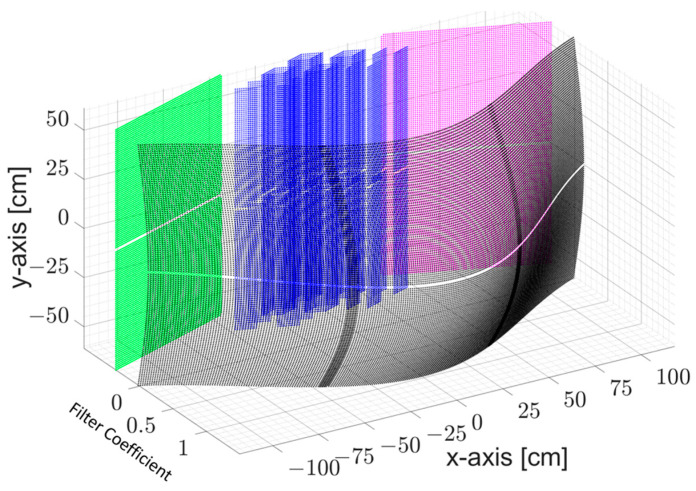
Kaiser filter with a coefficient of 4 over the virtual array aperture.

- Calibration: Signal processing errors emerge on a radar image as a high background level, indicating a noise level. To reduce the noise level, a calibration procedure based on [9] is developed. The calibration procedure involves the first filter in the imaging algorithm and determines the quality of the radar image by compensating channel responses. If the calibration is not rigorously performed, the quality of the radar image can distinctly degrade. More details are provided in Section 2.4.2.
- Hamming Filter: The Hamming filter is a well-known and frequently employed filter in radar image applications [10]. In this application, the 1D Hamming filter is used to suppress the range side lobes that lead to degradation in the quality of the radar image.
- Kaiser Filter: Virtual elements overlay the virtual aperture of the MIMO array. The abrupt end of virtual elements at the end of the virtual aperture can also degrade image quality, and so further smoothing might be required. The exact choice of the window coefficient will, of course, influence the lateral focusing quality of the array. This includes the side-lobe level as well as the resolution of the image. A suitable coefficient for the Kaiser filter is determined after the visibility comparison of the object, which is reconstructed for different coefficient values [11]. A coefficient of 4 has been chosen (Figure 8).

Once the virtual elements are placed at a spatial domain closer to the edges of the virtual aperture, their corresponding complex data within the frequency range are multiplied with a value closer to zero. If virtual elements are formed at a narrower virtual array aperture, the corresponding complex data are multiplied by one.

- Virtual Element Redundancy Filter: The illumination of the virtual array aperture is generally not flat and hence can include abrupt changes. Its smoothing is thus necessary to avoid abrupt illumination changes and preserve the image quality. The flattening of the aperture is thus performed by normalizing the scattering coefficient values by means of a number of antenna pairs whose virtual elements form at the same spatial position within the virtual array aperture.

The filter first determines all the combinations of transmitter and receiver antenna pairs that contribute to the same spatial position over the x-y-axes within the virtual array aperture. Next, the determined antenna pairs count $CF\left(r_{{n,m}_{VE}}\right)$ is used to normalize the corresponding Kaiser-filter-applied scattering coefficients $s_{RF}\left(r_{n_{Tx}},r_{m_{Rx}},f_{k}\right)$, as given in Equation (1). This calculation is processed within the virtual array aperture:

(1) $$s_{RF}\left(r_{n_{Tx}},r_{m_{Rx}},f_{k}\right)=\frac{s_{KF}\left(r_{n_{Tx}},r_{m_{Rx}},f_{k}\right)}{CF\left(r_{{n,m}_{VE}}\right)}\;,$$

where $r_{n_{Tx}}$ is the position of the *n*-th transmitter antenna, $r_{m_{Rx}}$ is the position of the m-th receiver antenna, $r_{{n,m}_{VE}}=\left(r_{n_{Tx}}+,r_{m_{Rx}}\right)/2$, and $f_{k}$ is the frequency value.

**Figure 9:**
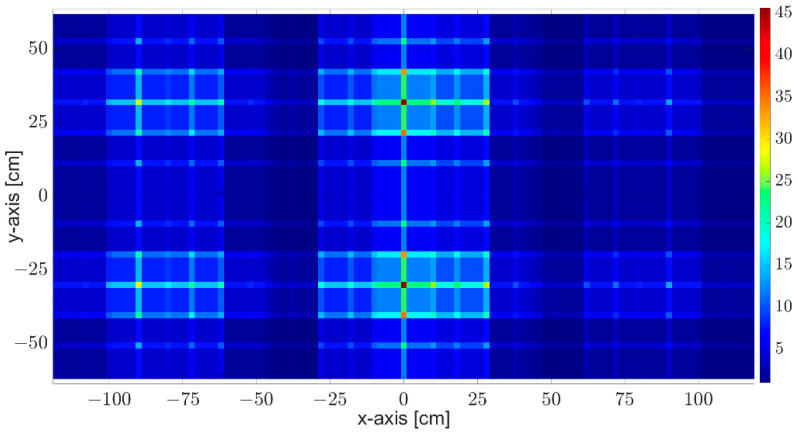
Redundancy filter coefficients represent how many virtual elements are co-located at the same spatial domain over the x-y virtual array aperture.

Figure 9 represents the redundancy filter coefficient map, $CF\left(r_{{n,m}_{VE}}\right)$, in Equation (1). This map reveals the co-located virtual elements’ count over the virtual array aperture on the x-y-axes.

- Velocity Estimation and Compensation: During the acquisition, the illuminated person is in motion. Although a single transmission time is below a millisecond, the total measurement time is 70 ms due to the time-division multiplexing mode of 176 transmitter antennas for each sub-system. This movement finally results in a shift in the spatial domain and needs to be compensated using a velocity compensation filter.

The *Velocity estimation* algorithm calculates the velocity of a walking person based on an additional single-channel radar sensor operating at 80 GHz. First, static clutter is removed from the data measured by the speed measurement system. Then, 2D fast Fourier transform (FFT) of up and down chirp data is taken to obtain range-velocity maps. Afterwards, these two maps are pointwise multiplied with each other to enhance the dynamic range. The center of gravity of the spectrum is calculated to obtain the velocity of the person. Finally, the object’s velocity $v_{o}$ is conveyed to the velocity compensation filter, as shown in Figure 7.

**Figure 10:**
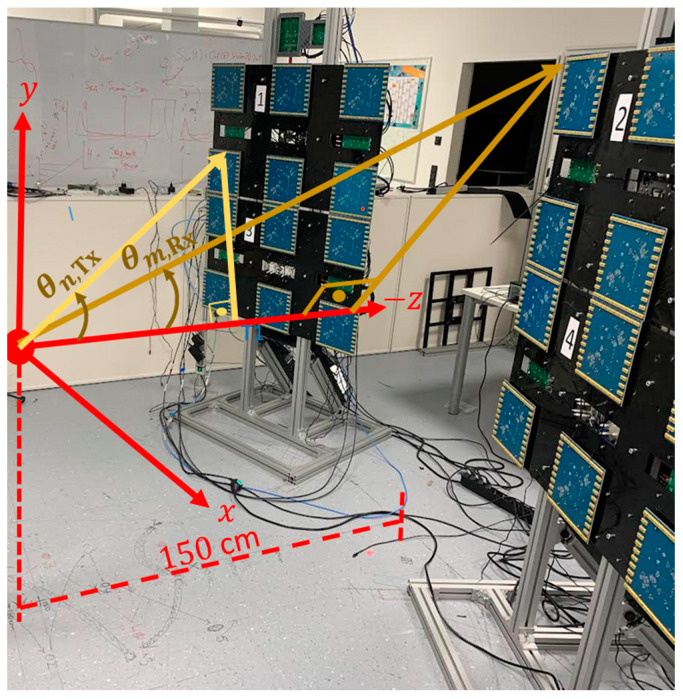
Transmission $\theta_{n,Tx}$ and reception $\theta_{m,Rx}$ angles in the measurement scene.

Regarding Velocity compensation algorithm, in the filter, the transmission $\theta_{n,Tx}$ and reception $\theta_{m,Rx}$ angles, as shown in Figure 10, have to be first calculated for every transmitter and receiver antenna between the z-axis and line segments from the center of the region of interest to the corresponding transmitter and receiver antennas, respectively.

For each transmitter/receiver pair, the total range displacement is calculated as follows:

(2) $${∆}_{total}={∆}_{Tx}+{∆}_{Rx}\;,$$

where range displacements ∆_Tx_ and ∆_Rx_ for transmitter and receiver antennas, respectively can be obtained as follows:

(3) $$\begin{array}{c} {∆}_{Tx}=v_{o}\cdot \left(n-1\right)\cdot t_{SingleTime}\cdot \cos\theta_{n,Tx}\;,\\ {∆}_{Rx}=v_{o}\cdot \left(m-1\right)\cdot t_{SingleTime}\cdot \cos\theta_{m,Rx}\;, \end{array}$$

where *t*_SingleTime_ is the transmission time for a single transmitter antenna, and n and m are the transmitter and receiver antenna indexes, respectively. Finally, velocity compensation coefficients can be calculated as follows:

(4) $${CF}_{velComp}\left(n,m,k\right)=e^{-2i\cdot {∆}_{total}\cdot \pi \cdot f_{k}/c}\;,$$

where *c* is the speed of the electromagnetic wave in free space. Velocity compensation coefficients of Equation (4) are finally multiplied by the redundancy-applied signal of Equation (1).

#### 2.4.2. Calibration Procedure

For a reliable reconstruction of the reflectivity map, cross-coupling and channel response effects in the measured signal need to be compensated. The cross-coupling $r_{cc}$ influences phase and amplitude in the measured signal, thereby deteriorating the radar image. Aside from the cross-coupling, the effect of channel responses for all transmitter and receiver antennas belonging to the MIMO imaging system is also prominent in the measurement signal. To achieve the reliable reconstruction of the reflectivity map with minimal noise, it is crucial to minimize cross-coupling and channel responses in the measured signal. The proposed calibration method succeeds in two steps. First, a channel response of an active calibration unit (ACU) is obtained. This ACU is an equivalent board that composes the imaging system (Figure 12) but is used with only one transmitter and one receiver channel. This first step involves a metal plate measurement and subsequently the calculation of the channel response, based on these measured scattering coefficients. After this, channel responses belonging to the MIMO imaging system are obtained by performing first a transmission measurement between the ACU and the MIMO imaging system and then calculating these channel responses in consideration of the channel response of the ACU and this transmission scattering coefficient.

**Figure 11:**
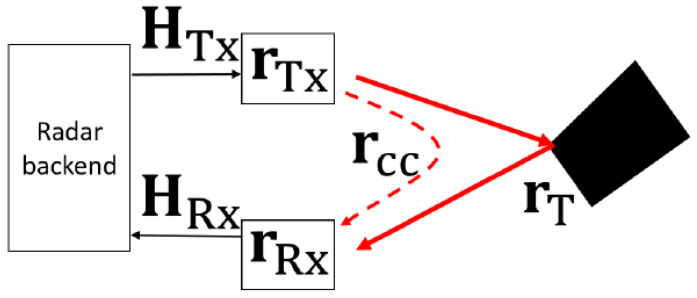
The measured signal consists of channel responses of transmitter and receiver antennas, coupling effect (dotted red arrow), and target reflection (solid line red arrow).

As shown in Figure 11, the measurement $s_{meas}(r_{n_{Tx}},r_{m_{Rx}},f_{k})$ and background $s_{bg}(r_{n_{Tx}},r_{m_{Rx}},f_{k})$ signals can be represented as follows:

(5) $$s_{meas}\left(r_{n_{Tx}},r_{m_{Rx}},f_{k}\right)=s_{target-meas}\left(r_{n_{Tx}},r_{m_{Rx}},f_{k}\right)\cdot H_{n_{Tx}}\cdot H_{m_{Rx}}+s_{cc}\left(r_{n_{Tx}},r_{m_{Rx}},f_{k}\right)\cdot H_{n_{Tx}}\cdot H_{m_{Rx}}\;,$$

(6) $$s_{bg}\left(r_{n_{Tx}},r_{m_{Rx}},f_{k}\right)=s_{cc}\left(r_{n_{Tx}},r_{m_{Rx}},f_{k}\right)\cdot H_{n_{Tx}}\cdot H_{m_{Rx}}\;,$$

where

(7) $$s_{cc}\left(r_{n_{Tx}},r_{m_{Rx}},f_{k}\right)=A_{cc}\left(r_{n_{Tx}},r_{m_{Rx}},f_{k}\right)\cdot e^{-i\frac{2\pi f_{k}r_{cc}}{c}}\;,$$

and where $s_{target-meas}\left(r_{n_{Tx}},r_{m_{Rx}},f_{k}\right)$ is the back-reflected target response; $H_{n_{Tx}},H_{m_{Rx}}$ are the channel responses for *n*-th transmitter and m-th receiver antennas, respectively; and $s_{cc}\left(r_{n_{Tx}},r_{m_{Rx}},f_{k}\right)$ is the cross-coupling signal introduced with an amplitude of $A_{cc}\left(r_{n_{Tx}},r_{m_{Rx}},f_{k}\right)$. The background signals only consist of the cross-coupling signals for every transmitter/receiver pair due to measurements performed in an empty space. The cross-coupling signal with its components is minimized by considering the background signal in one direction. To isolate the channel responses $H_{n_{Tx}},H_{m_{Rx}}$, a well-known scene has to be chosen which can be modeled without numerical effort. For the given configuration, a reflective setup is not capable of covering the different orientation angles of the modules. Therefore, an active calibration method, based on an external “repeater”, was used to act as an artificial point scatterer.

**Figure 12:**
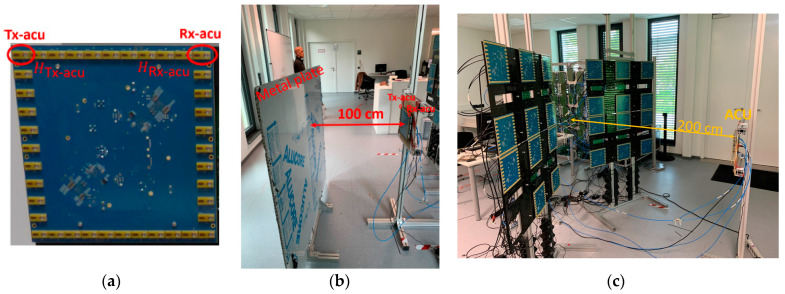
Active calibration method. (**a**) An active calibration pair is employed on the ACU; (**b**) metal plate measurement to obtain a channel response of this active calibration pair; (**c**) transmission measurement between the active calibration pair and the proposed imaging system.

With the proposed calibration method, an external transceiver, namely the active calibration unit (ACU), is used as shown in Figure 12a. A pair of transmitter Tx-ACU and receiver Rx-ACU antennas is assigned as an active calibration pair on the ACU. In the active calibration method, channel responses $H_{Tx-ACU}$, $H_{Rx-ACU}$ for transmitter Tx-ACU and receiver Rx-ACU antennas, respectively, should first be obtained. To improve SNR in the calibration signal, 10 sequential measurements with a metal plate, positioned 100 cm away from the ACU as shown in Figure 12b, are performed and then averaged. To minimize the cross-coupling, 10 sequentially measured and subsequently averaged background scattering coefficients are subtracted from the metal plate measurements. The measured signal on the ACU can be expressed by the following:

(8) $$s_{meas-ACU}\left(f_{k}\right)=H_{Tx-ACU}\cdot H_{Rx-ACU}\cdot A_{meas-ACU}\;,$$

where $A_{meas-ACU}$ is the propagation path between the metal plate and the active calibration pair on the ACU. The channel response of the ACU can be calculated as follows:

(9) $$H_{ACU}=\frac{H_{Tx-ACU}\cdot H_{Rx-ACU}\cdot A_{meas-ACU}}{A_{sim-ACU}}=H_{Tx-ACU}\cdot H_{Rx-ACU}\;,$$

where $A_{sim-ACU}$ is the simulated propagation path between the active calibration pair and the metal plate, and can be described as follows:

(10) $$A_{sim-ACU}=e^{-i\frac{2\pi f_{k}}{c}\left(\left|r_{mp}-r_{Tx-ACU}\right|-\left|r_{mp}-r_{Rx-ACU}\right|\right)}\;,$$

where $r_{mp}$ is the metal plate position, $r_{Tx-ACU}$ and $r_{Rx-ACU}$ are positions of the transmitter Tx-ACU and receiver Rx-ACU antennas, respectively.

The next step is transmission measurement, as shown in Figure 12c. Here, the center of the ACU is positioned 200 cm away from the center of the array aperture. The Tx-ACU illuminates all the receiver antennas $r_{m_{Rx}}$ on the imaging system, whereas the Rx-ACU receives from transmitter antennas $r_{n_{Tx}}$ on the imaging system. Because there is a distance of 200 cm between the imaging system and the ACU, the transmission scattering parameters are free of the cross-coupling influence.

The transmission scattering parameters, measured between $r_{n_{Tx}}$ and Rx-ACU, and $r_{m_{Rx}}$ and Tx-ACU, respectively, can be represented as follows:

(11) $$\begin{array}{c} s_{meas-Tx\&Rx-ACU}\left(n_{Tx}\right)=H_{n_{Tx}}\cdot H_{Rx-ACU}\cdot A_{meas-Tx\&Rx-ACU}\left(n_{Tx}\right)\;,\\ s_{meas-Rx\&Tx-ACU}\left(m_{Rx}\right)=H_{m_{Rx}}\cdot H_{Tx-ACU}\cdot A_{meas-Rx\&Tx-ACU}\left(m_{Rx}\right)\;, \end{array}$$

where $A_{meas-Tx\&Rx-ACU}\left(n_{Tx}\right)$ and $A_{meas-Rx\&Tx-ACU}\left(m_{Rx}\right)$ are the propagation paths from the *n*-th transmitter antenna on the imaging system to the Rx-ACU, and from the Tx-ACU to the m-th receiver antenna on the imaging system, respectively. In consideration of antenna positions on the imaging system and ACU in the measurement scene, channel responses belonging to these transmission measurements can be calculated by considering simulation propagation paths as follows:

(12) $$\begin{array}{c} H_{n_{Tx}\&Rx-ACU}\left(n_{Tx}\right)=\frac{H_{n_{Tx}}\cdot H_{Rx-ACU}\cdot A_{meas-Tx\&Rx-ACU}\left(n_{Tx}\right)}{A_{sim-Tx\&Rx-ACU}\left(n_{Tx}\right)}=H_{n_{Tx}}\cdot H_{Rx-ACU}\;,\\ H_{m_{Rx}\&Tx-ACU}\left(m_{Rx}\right)=\frac{H_{m_{Rx}}\cdot H_{Tx-ACU}\cdot A_{meas-Rx\&Tx-ACU}\left(m_{Rx}\right)}{A_{sim-Rx\&Tx-ACU}\left(m_{Rx}\right)}=H_{m_{Rx}}\cdot H_{Tx-ACU}\;,\; \end{array}$$

where the simulation propagation paths can be described as follows:

(13) $$\begin{array}{c} A_{sim-Tx\&Rx-acu}\left(n_{Tx}\right)=e^{-i\frac{2\pi f_{k}}{c}\left(\left|r_{n_{Tx}}-r_{Rx-ACU}\right|\right)}\;,\\ A_{sim-Rx\&Tx-acu}\left(m_{Rx}\right)=e^{-i\frac{2\pi f_{k}}{c}\left(\left|r_{m_{Rx}}-r_{Tx-ACU}\right|\right)}\;, \end{array}$$

Channel responses of the imaging system for every antenna pair can be finally calculated as follows:

(14) $$H_{Tx\&Rx}\left(n,m\right)=\frac{H_{n_{Tx}\&Rx-ACU}\;.\;H_{m_{Rx}\&Tx-ACU}\;\;}{H_{ACU}}=H_{n_{Tx}}\cdot H_{m_{Rx}}\;.$$

To avoid numerical artefacts from the direct division of noisy measured target signal and channel responses, calibration coefficients are obtained as follows:

(15) $${CF}_{Tx\&Rx}\left(n,m\right)=\frac{\bar{\left(H_{Tx\&Rx}\left(n,m\right)\right)}}{\left({\left|H_{Tx\&Rx}\left(n,m\right)\right|}^{2}+{factor}_{smoothing}^{2}\right)}\cdot {factor}_{amplitude}\;.$$

The component factor_smoothing_ is added to avoid overweighting of small amplitudes arising from the division by a measured quantity, whereas factor_amplitude_ helps with tuning amplitude values of the radar imaging system into the necessary range for the ATD algorithm.

Finally, the pure object signal is obtained for every transmitter/receiver pair as follows:

(16) $$s_{target-meas}\left(r_{n_{Tx}},r_{m_{Rx}},f_{k}\right)=\left[s_{meas}\left(r_{n_{Tx}},r_{m_{Rx}},f_{k}\right)-s_{bg}\left(r_{n_{Tx}},r_{m_{Rx}},f_{k}\right)\right]\cdot {CF}_{Tx\&Rx}\left(n,m\right).$$

### 2.5. Imaging System Characterization

Enhancing the imaging performance of a radar system is an imperative necessity, which is closely linked to the system’s attributes, including the level of illuminated power density, the signal-to-noise ratio (SNR) of the transmitted signal, and the point spread function.

#### 2.5.1. Point Spread Function Characterization

Since measured scattering parameters were not obtained during the system development, the PSF characteristics are studied using simulation data. To investigate the PSF quality, a point-like target is placed at the center of the coordinate system and the proposed MIMO array is positioned 150 cm away from the focal point as given in Figure 4. Data collected in the simulation is then focused using the imaging algorithm stated in Section 2.6. 

**Figure 13:**
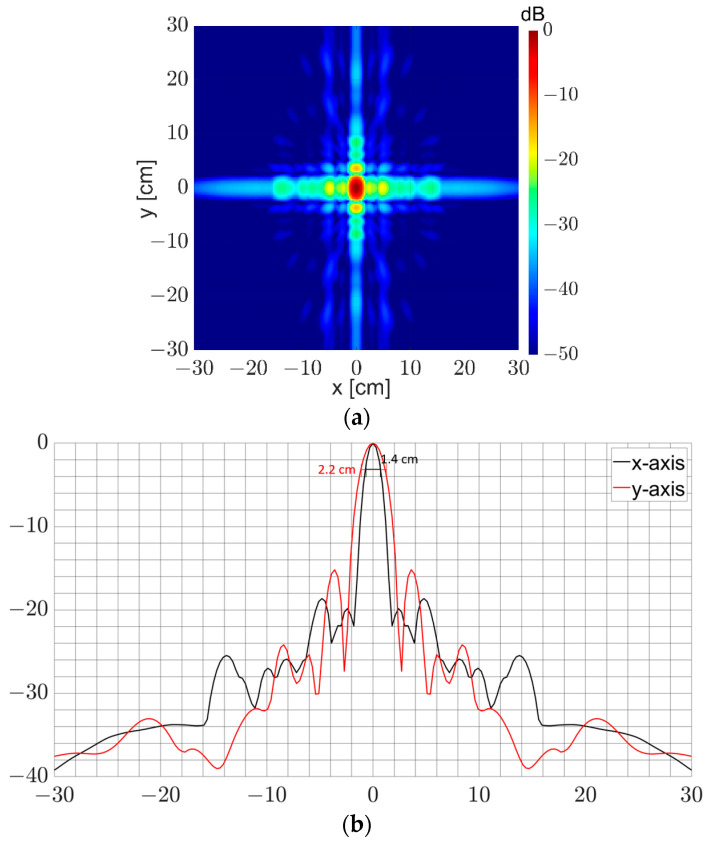
(**a**) Normalized 2D PSF on x-y-axes; (**b**) normalized 1D PSF along with x- and y-axis.

We note in Figure 13a that pedestal side lobes form in the horizontal and vertical directions where the shadowing effect is the strongest within the virtual array. The maximum side-lobe levels are about −18.6 and −15.1 dB along the x-y-axes, respectively, which are low enough to obtain a high-contrast radar image. It is clear that the ultra-wide frequency band helps squeeze the PSF around the main beam residing by virtue of strong interference of transmitted waves. This strong interference lessens at the larger x-y-axes in Figure 13b. The simulated resolutions of the system shown in Figure 13 are 1.4 cm and 2.2 cm.

#### 2.5.2. Signal-to-Noise Ratio Measurement

Signal-to-Noise-Ratio (SNR) is defined as the ratio of the power of the desired signal to the power of the random noise component. In a static radar measurement, the measured complex value at each range bin should be constant with time. Assuming the white Gaussian receiver noise, the noise power can be calculated using the variance of the complex measured signal at the range bin under test over time. A sequence of calibration measurements in a static environment can therefore be used to calculate the SNR of every transmitter and receiver as the ratio of the square of the mean value at the range bin under test and the corresponding variance:

(17) $$\begin{array}{c} {SNR}_{n_{Tx}}=10\cdot {log}_{10}\left(\frac{\mu_{n_{Tx}}^{2}}{\sigma_{n_{Tx}}^{2}}\right)\;dB,\\ {SNR}_{m_{Rx}}=10\cdot {log}_{10}\left(\frac{\mu_{m_{Rx}}^{2}}{\sigma_{m_{Rx}}^{2}}\right)\;dB, \end{array}$$

where *μ_n_*_Tx_ and *μ_m_*_Rx_ are the signal means for the *n*-th transmitter and for the *m*-th receiver, respectively, *σ_n_*_Tx_ and *σ_m_*_Rx_ are the standard deviations of the noise over time for corresponding transmit (respectively receive) antennaUsing the same measurement setup in Figure 12c, the sequential 10 transmission measurements are performed between the transmitter antenna on the system and ACU receiver antenna, and the receiver antenna on the system and ACU transmitter antenna. In total, 880 transmission measurements are obtained from 528 receiver and 352 transmitter antennas. Subsequently, the inverse Fourier transform of these measurements generates a range distribution in the spatial domain.

**Figure 14:**
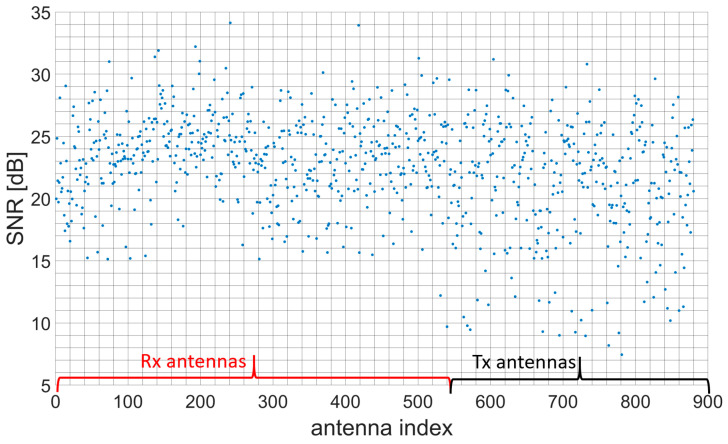
SNR values for transmitter and receiver antennas.

Figure 14 represents the SNR values obtained for every antenna. Most of the antennas are operational with SNR values greater than 15 dB. Nevertheless, there are 1 Rx and 9 Tx antennas with SNR values below 10 dB.

#### 2.5.3. Radiation Power

The power density transmitted by security scanners is addressed in a report [12], which indicates that, for frequencies between 2 and 300 GHz, millimeter-wave security scanners would be used, and the maximum power density level recommended is 10 W/m² for members of the public and 50 W/m² for exposed workers.

The frequency range transmitted from the microwave imaging system will be within 6.5 to 10.5 GHz. The regulations make it necessary to determine whether the transmitted power density is below the maximum threshold.

**Figure 15:**
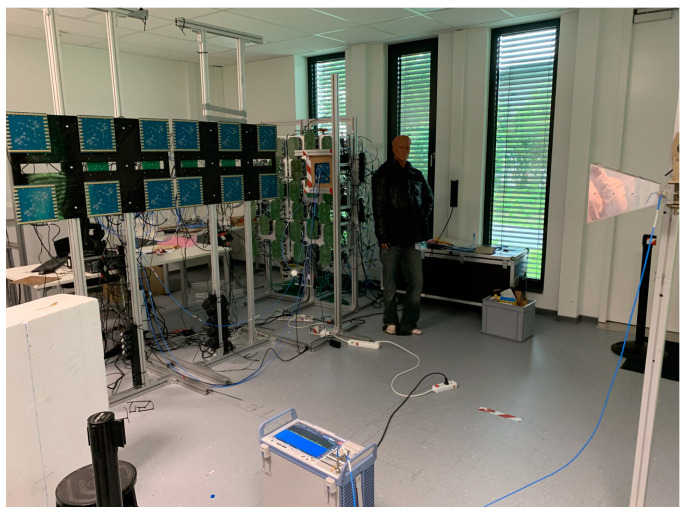
Signal measurements at 3 m.

The measurement is performed at three different ranges, such as 1 m, 2 m, and 3 m (Figure 15). One transmitter antenna is activated and continuously illuminates the measurement scene including the double ridge horn antenna. The polarizations of the double ridge horn antenna and Tx antenna on the imaging system are deliberately matched.

Received powers on the spectrum analyzer at 8.5 GHz at 1 m, 2 m, and 3 m are −66.4, −71.1, and −82.2 dBm, respectively. After considering the losses and receiver antenna gain, the power density S can be calculated for these three different ranges, resulting in 6.2897.10^−7^ W/m^2^, 2.1313.10^−7^ W/m^2^, and 0.1652.10^−7^ W/m^2^, respectively. 

The power densities obtained are dramatically lower than the power density level limit of 10 W/m² for members of the public [12], which means that the MIC system is compliant with European regulations and can be used in public locations.

Since the imaging system is thoroughly characterized and compliant with requirements, the next step is to perform the ATD based on an Artificial Intelligence (AI) algorithm.

### 2.6. Artificial Intelligence Algorithm for Automatic Detection on Radar Imagery

Data are the central elements of AI methods. Having large quantities of uality data is crucial to allow the convergence of learning and the correct measurement of performance during tests. As MIC is a prototype developed in a time-constrained project, only a few training data set measurement campaigns have been conducted. An operational system would require much more data.

All available, non-empty, and exploitable voxels (i.e., 3D pixels of the MIC image) have been processed and transformed into a common compressed 2D format with two channels. Each "voxel cut" has a size of (120, 100, 2). One channel represents the amplitude, whereas the other represents the relative distance of the brightest pixel (i.e., the closest in range). The format compresses each 3D voxel into a simpler individual 2-channel 2D image at the estimated center position of the subject. The compression results in virtually no information loss since most of the 3D voxel space comprises pure noise. The voxel cuts are used for training and testing the CNN-based classifier, which is discussed in the next section. 

A total of 15,251 usable voxel cuts with 83 different subjects were produced during the training phases. A Convolutional Neural Network (CNN)-based classifier was trained with four selected threats (AK47, middle-sized automatic rifle, small gun, and explosive belt mock-up), with confusers (umbrella, smartphone, metallic bottle, among other things), and without any object.

#### 2.6.1. MIC Convolutional Neural Network Classifier and Explainable Artificial Intelligence Description

- Convolutional Neural Network

A convolutional neural network (CNN, or ConvNet) is a class of artificial neural networks, most commonly applied to analyzing imagery [13,14]. CNNs are regularized versions of multilayer perceptrons, and use several convolution layers piled on top of each other (leading to the expression “deep learning”). CNNs use relatively little pre-processing compared to other image classification algorithms. This means that the network learns to optimize the filters (or kernels) using automated learning, whereas in traditional algorithms these filters are hand-engineered. This independence from prior knowledge and human intervention in feature extraction is a major advantage.

In a CNN, the input is a tensor with a shape: (number of inputs or batch size) × (input height) × (input width) × (input channels). After passing through a convolutional layer, the image becomes abstracted to a feature map, also called an activation map, with the following shape: (number of inputs) × (feature map height) × (feature map width) × (feature map channels). Convolutional layers convolve the input and pass its result to the next layer. They are ideal for data with a grid-like topology (such as images) as spatial relations between separate features that are taken into account during convolution and/or pooling. Among successive convolutional layers, the filters focus on very simple features, such as outlines, and grow in complexity and granularity on features that uniquely define the object.

Like other neural networks, a convolutional neural network is composed of an input layer, an output layer, and many hidden layers. These perform operations that modify the data in order to learn specific characteristics.

The four layers that constitute the most common building blocks of the intermediate layers of CNNs are a convolution layer, a normalization layer, an activation layer, and a reduction layer (pooling). Each stacking of these elementary layers is often called a “down-sampling layer”. A deep CNN network then comprises the sequence of several of these stages.

To perform a classification function, the network ends with one or more linear layers intended to distribute the learned patterns into different probabilities belonging to each of the output classes. The last layer of the architecture uses a classification layer such as softmax to generate the classification output. Classification is, therefore, carried out on a low-dimensional so-called “latent” space, which is found at the output of the last convolutional neuronal layer (output of down-sampling blocks).

For the MIC project, a fairly “classic” CNN deep learning architecture was chosen using five down-sampling blocks: convolution, normalization, activation, terminated with one fully connected layer, and a softmax layer that simply converts output class scores into a probability distribution of these classes (normalized between 0 and 1). Classic random data augmentations were applied to reduce overfitting by training the machine learning model on several slightly modified copies of existing data: x-flip voxels, ±20 cm x-translation (x-centered), ±10 cm translation in height, and ±4 cm translation in distance (centered).

- Explainable Artificial Intelligence module

Interpretability is the degree to which machine learning algorithms can be understood by humans. Machine learning models are often referred to as “black boxes” because their representations of knowledge are not intuitive and, as a result, it is often difficult to understand how they work. Interpretability techniques help to reveal how black-box machine learning models make predictions. In the context of deep neural networks, interpretability is more often referred to as “AI explainability” [15].

By revealing how various features contribute (or do not contribute) to predictions, interpretability techniques can help to validate that the model is using appropriate evidence for predictions.

**Figure 16:**
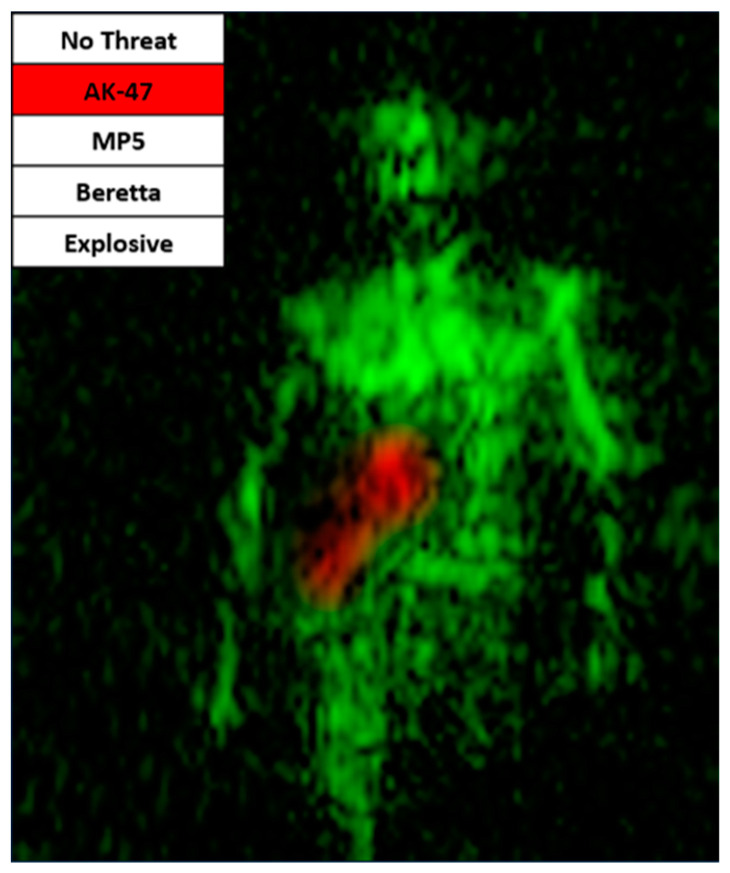
Explainable AI example on MIC AK47 detection: red spot indicates the main body area contributing to threat detection by the AI classifier.

We have used LIME [16] and Grad-CAM [17] techniques to highlight the regions of an image that contribute most to the predictions of the image classification network. An example of a result is shown in Figure 16. These techniques clearly help to interpret decisions. For instance, it showed that explosive threat detection was mainly determined based on a deviation of the “natural” belly region signature caused by the dielectric masking effect of the explosive belt and the abnormal presence of scattered metal parts. During AI development, it also helped us to improve the model by recognizing whether the classifier was trustworthy or not when comparing the highlighted region with the available ground truth. 

#### 2.6.2. MIC Classification Performance Assessment on Static Targets

**Figure 17:**
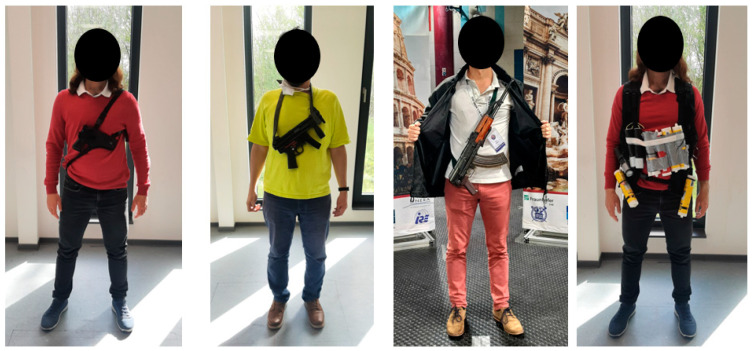
Threats used for MIC performance measurement scenarios (small gun, semi-automatic MP5 also called big gun, AK-47, explosive belt with nuts and bolts).

MIC Classification Performance measurement was conducted with several threats of interest with different sizes and shapes (Figure 17).

**Figure 18:**
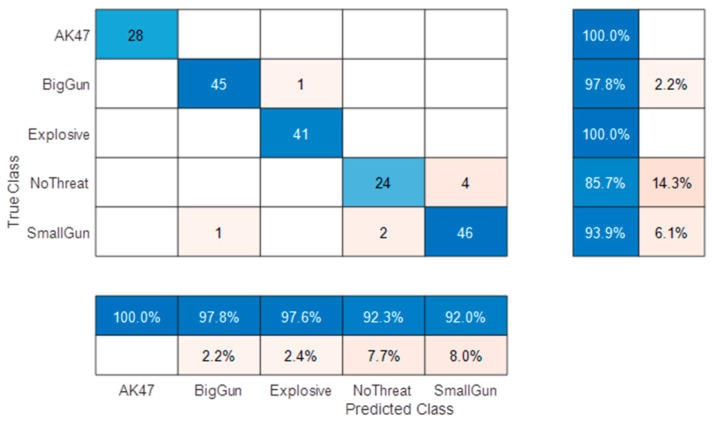
Confusion matrix with random separation in static 5 classes classification.

Before the field trial, a first classification performance analysis was conducted on a reduced dataset using only static target measurements (20 subjects). A random separation of 841 train and 192 independent test voxels among all subjects was performed and led to the confusion matrix shown in Figure 18.

Accuracies (and prediction errors) are presented on the right of the confusion matrix (mean accuracy = 95.8%). The probabilities of correct (and false) declarations are presented at the bottom.

The confusion matrix shows an excellent class separation (residual errors due to the small gun, which is at the limit of target size detection), which confirms the chosen CNN as a good potential candidate for ATD. However, such a confusion matrix is useful for classifier architecture selection and optimization but not sufficient to infer a fair performance evaluation with independent subject separation from train and test measurements.

**Figure 19:**
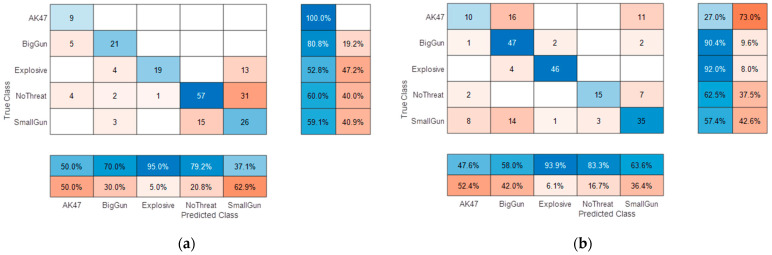
5 classes confusion matrices with: (**a**) Test subjects #1, #2, #3, and #4 and training on all other subjects; (**b**) test subjects #7, #8, #14, and #20 and training on all other subjects.

It is important to check that the performance of a network is not biased by the subjectivity of the dataset it is trained on. The best way to ensure this is to separate the subject selection between the Train and Test subsets. Figure 19 shows the confusion matrices obtained using two different separations among the 20 available subjects.

These two confusion matrices show some similarities (e.g., probabilities of declaration) and major performance discrepancies (i.e., accuracies). In both cases, the overall performance is rather low (mean accuracy ~65%).

The high error variance is also due to the rather “low” subject cardinality (and the difference in the cardinality of available threat data between the two tests). However, both show quite good and consistent results for the “Explosive” declaration (~95%).

The missed alarm on the “NoThreat” declaration is mainly due to small gun targets, which correspond to the detectable size limit, but this poor performance was expected due to the rather low signature of such a threat (leading to low confidence in the declaration). Natural confusion between “BigGun” and “AK47” was also expected as weapons with similar sizes, which may lead to similar signatures.

**Figure 20:**
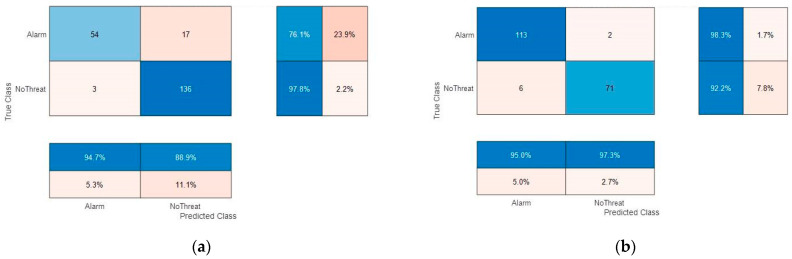
2 classes confusion matrices with: (**a**) Test subjects #1, #2, #3, and #4 and training on all other subjects; (**b**) test subjects #7, #8, #14, and #20 and training on all other subjects.

The performance may be further studied by reducing the problem to a binary classification: “big” threat alarm (AK47, BigGun, and Explosive) versus none. Corresponding confusion matrices are shown in Figure 20. The overall performance is slightly more consistent with a correct mean accuracy of > 90%.

With these static laboratory tests validating the processing chain, the complete system was transported to Rome, Italy, to be tested at Anagnina metro station (Rome, Italy).

## 3. Performance Evaluation

### 3.1. Live Demonstration Setup

The MIC imaging system was integrated within a dedicated area of a public metro station in Rome as part of the field trial and system demonstration of DEXTER. The integration phase was conducted in the first week of May 2022, followed by a demonstration for representatives of political, safety and economic institutions, customers, experts, and journalists at the end of the month.

**Figure 21:**
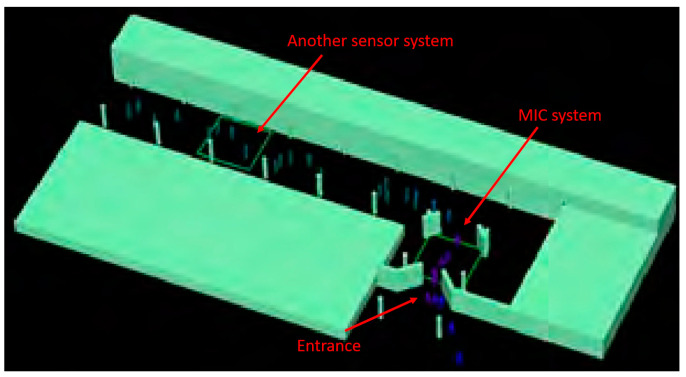
Layout of the measurement scene in Anagnina metro station. Passengers first have to pass through the MIC system.

The MIC system is built as a walkthrough solution. Passengers, entering the measurement scene as shown in Figure 21, must first pass through the MIC system. The resultant radar image is processed and potential threats are automatically identified and characterized. The DEXTER prototype is subsequently informed about the threat obtained from the MIC system to automatically initialize other sensor systems. Thus, the MIC system is an essential component in the DEXTER system prototype.

**Figure 22:**
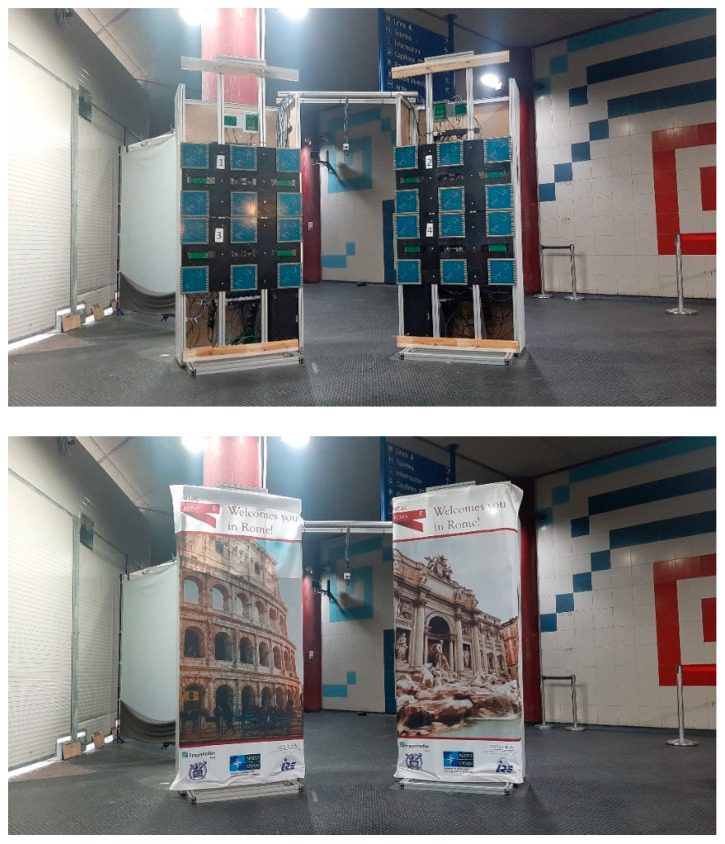
MIC implementation in Rome Anagnina metro station without and with advertising panels hiding antennas.

The final appearance of the MIC system is presented in Figure 22.

**Figure 23:**
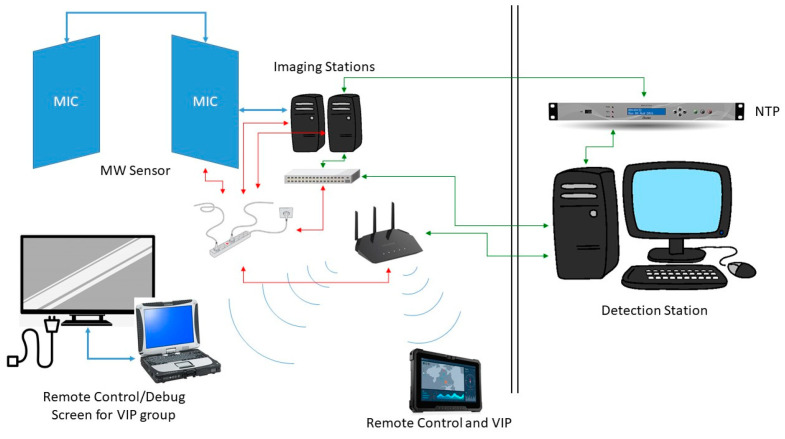
Synchronization of the MIC imaging system is accomplished via an Ethernet connection to the NTP server (Green arrows are datalink connections, red arrows are electric power ones).

The MIC imaging system was integrated into the DEXTER prototype via an NTP server to which two other sensor systems are connected, as shown in Figure 23. It is worth mentioning that the MIC system is set to be remotely controlled via laptop.

### 3.2. Field Trial Results

The MIC system performed experiments by discreetly scanning 550 people carrying different treatment objects under their jackets: 189 volunteers had a threat (weapon or explosive belt), 203 others had civilian objects (confusers), and 158 had no threat or object.

These volunteers participated in a blind test scenario covering different cases: either one by one, or four people walking in a row, or two people side by side. All the volunteers went through the MIC observed area with a natural walk and trajectory at a typical walking speed (~2 m/s).

The MIC system revealed a success rate of around 95% in these measurements, but performance can be defined in different ways (depending on the operational concept of operation):

- 92.6% of threat detection and identification;
- 93% of threat detection;
- 94% of objects concealed under clothes detection;
- 96% of big concealed object detection (if we do not take into account the small gun cases that were at the limit of the system in terms of target size).

Table 2 lists the MIC fail cases. We can observe that MIC confuses an explosive belt with a backpack carried on the front, which could be mitigated by a video camera and/or security agents. We can also conclude that most of the false positives and negatives concern small gun scenarios that are the identified limit of the system and objectives (due to threat size regarding resolution and image quality).

**Figure 24:**
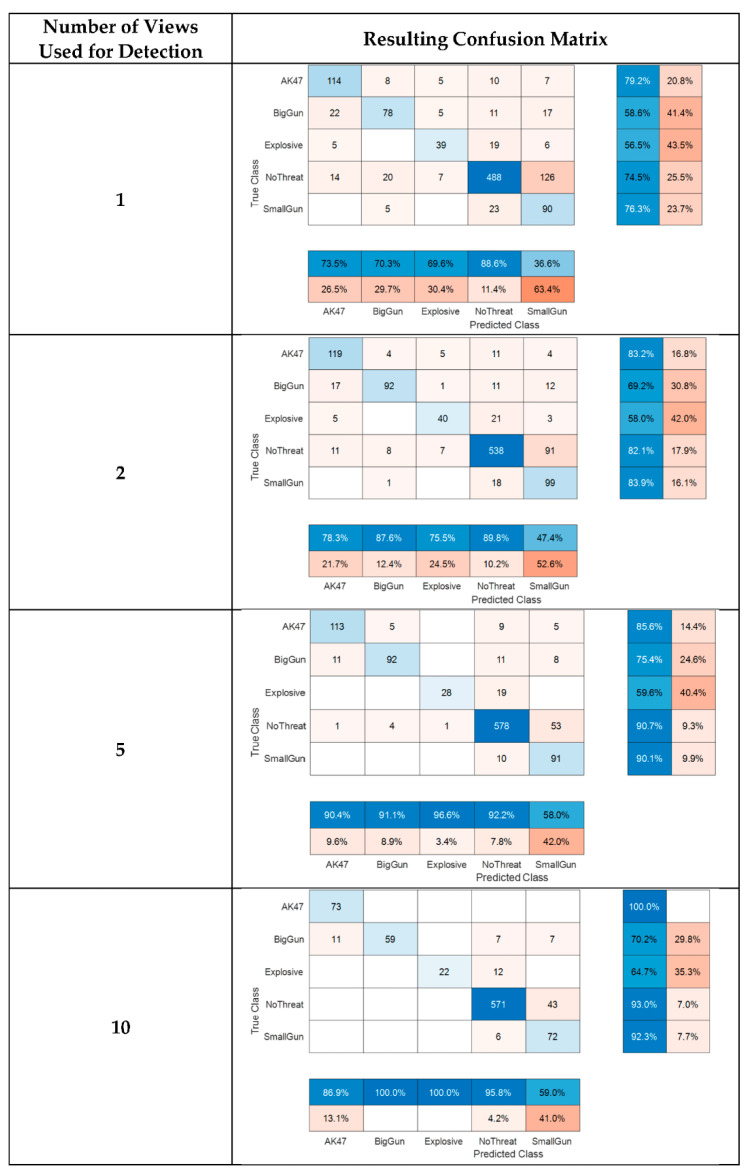
Post-field trial confusion matrices on field trail data, using simulated accumulation of 1 to 10 detections.

**Figure 25:**
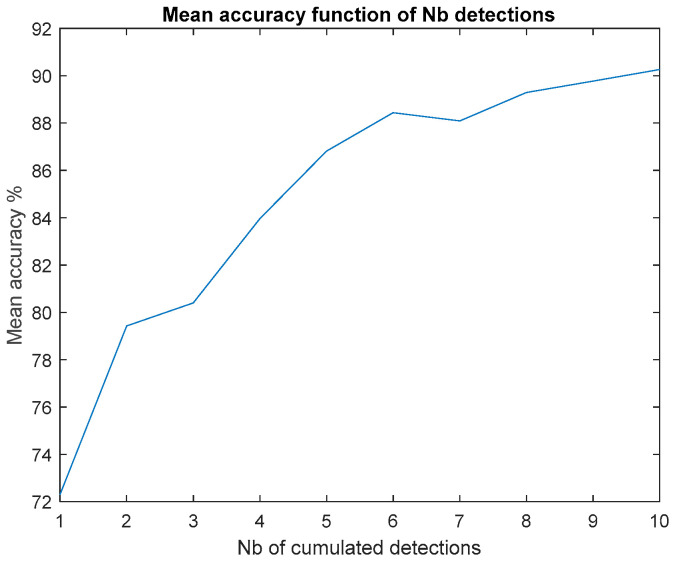
Post-field trial mean accuracy versus simulated detection accumulation.

We were able to simulate performances that could be obtained with a higher acquisition frame rate by combining several paths of the same subject carrying the same threat. The positive effect brought on by cumulating the individual detections is Figure 24 and Figure 25.

These results confirm the utility of increasing the image frame rate for a future operational system.

Field trial results must be weighted as the tests carried out did not present a complete separation of the subjects between the test and learning space due to the relatively limited number of volunteers. To mitigate this issue, we undertook post-trial data reprocessing with a careful check of the subjects’ identity according to the available ground truth.

**Figure 26:**
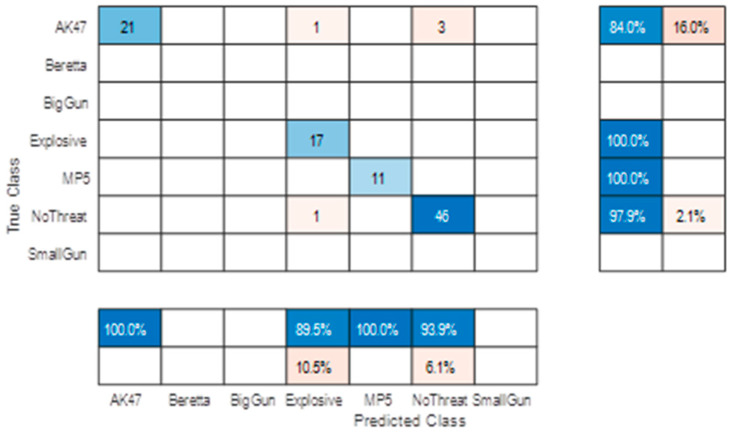
Post-field trial confusion matrix on independent subject selection (decision made on two successive detections).

We then retrained and tested the AI on fully independent datasets. We also added two classes with threats only available in laboratory experiments (big gun and small gun other than Beretta). We obtain the confusion matrix shown in Figure 26 (decision on two successive frames).

Overall performance increases in line with the number of training subjects and number of cumulated detections, and confirms the announced ~95% accuracy for major threat detection (decision on two successive detections).

**Figure 27:**
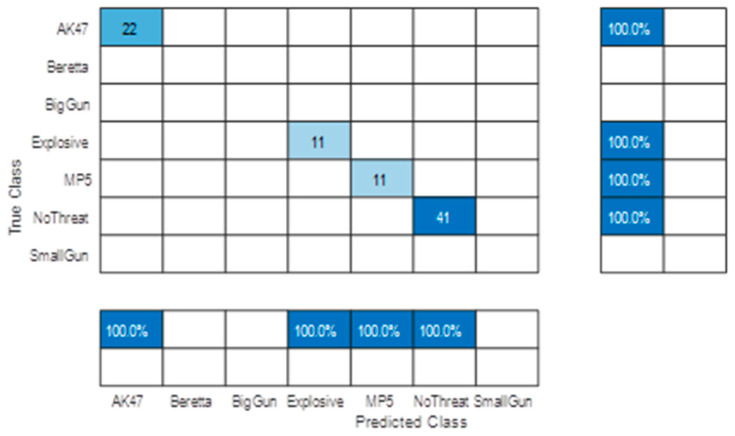
Post-field trial confusion matrix on independent subject selection (decision made on four simulated successive detections).

Moreover, this reprocessing enables us to artificially simulate an increase in the system frame rate by combining several passages of the same subject (with of course the same threat conditions). The positive impact of doubling the frame rate is clearly shown in the confusion matrix of Figure 27.

## 4. Perspectives for Operational Sensors: Wider Door and Wall Configuration

The MIC prototype exhibits the ability to automatically detect threats concealed under the clothing of pedestrians in motion, without interrupting their movement. This is accomplished using COTS low-cost components that comply with EU regulations concerning civilian health in the presence of electromagnetic sources.

However, certain aspects require improvement before transferring the prototype to a higher maturity level and proposing a COTS solution to end-users. One such aspect pertains to discretion, particularly, since MIC is built as a walkthrough solution, with respect to the space between the right and the left panels. While an 87 cm passage is typical of a standard office door, it may prove too narrow for individuals carrying large wheeled suitcases or for those with disabilities, such as those using wheelchairs. Widening the passage may be possible, but it could potentially lead to a loss in performance.

**Figure 28:**
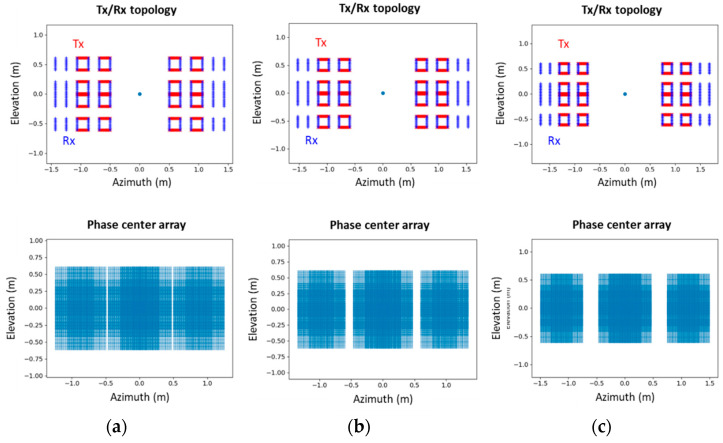
Sensor antenna array and corresponding phase center array in the azimuth/elevation plane for different spacing between MIC left and right panels: (**a**) 1 m; (**b**) 1.2 m; (**c**) 1.5 m in door configuration.

**Figure 29:**
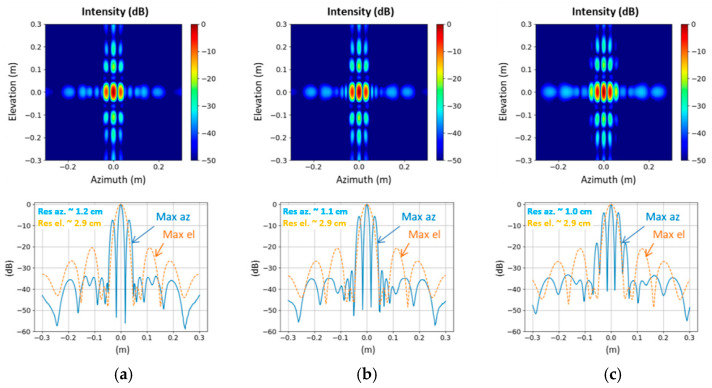
Maximum intensity in the azimuth/elevation plane and cuts in azimuth and elevation direction in dB (normalized) for different spacing between MIC left and right panels: (**a**) 1 m; (**b**) 1.2 m; (**c**) 1.5 m in door configuration.

Simulations of possible artefacts encountered when widening this door passage, with a similar system configuration to the one used for the field trial in Rome, are presented in Figure 28 for three door widths: 1 m, 1.2 m, and 1.5 m. Figure 29 shows the maximum intensity in the azimuth/elevation plane obtained for a point-like target located 1.5 m from the door passage.

When increasing the width of the gap between the left and right panels, the antenna dimension in the azimuth axis is increased as well. We can then observe an improvement in the resolution in the azimuth direction. However, gaps appear in the phase center array, which leads to a rise of the side lobes level in the azimuth direction.

Inevitably, this will lead to a degradation of image quality. However, the impact on the performance of the ATD is yet to be assessed. Since it is based on a CNN algorithm, learning on a degraded image base may still produce an acceptable threat detection rate. It would be interesting to investigate this point in future work.

**Figure 30:**
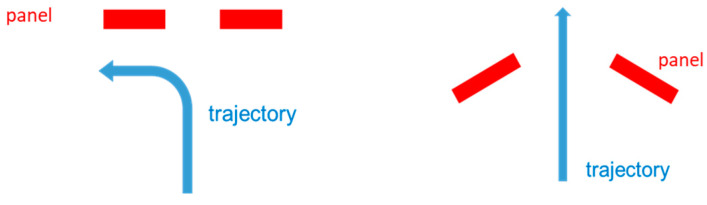
Two different configurations for MIC: (left) wall and (right) door.

It would be of significant interest to analyze the performance of the ATD algorithm when the system is configured in the "wall" layout. This particular configuration is characterized by a higher level of discretion compared to the door configuration and does not impose any constraints on the flow of passengers. However, one significant drawback of this configuration is that people would be required to turn a corner located in front of the system (as illustrated in Figure 30), which may result in a loss of weapon visibility and detection. It is worth noting, however, that this configuration can also provide an opportunity for people to be viewed from the side or the back, particularly when two wall sensors are installed in a corner.

**Figure 31:**
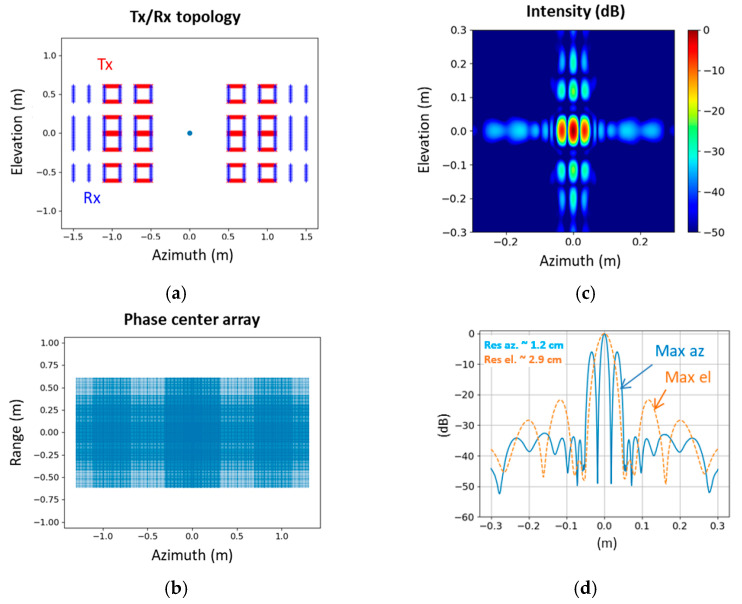
Wall configuration with (**a**) Sensor antenna array in the azimuth/elevation plane for wall configuration. (**b**) Corresponding phase center array. (**c**) Maximum intensity in the azimuth/elevation plane in dB (normalized). (**d**) Maximum intensity levels in the azimuth or elevation direction in dB (normalized).

The results of applying the back-projection algorithm in the wall layout are presented in Figure 31 for a point-like target. Notably, the virtual antenna array displays a full array without any gaps in comparison to the door configurations illustrated in Figure 29. Hence, it would be worthwhile to evaluate the performance of a newly trained ATD algorithm on this particular sensor configuration, similar to the approach taken with large distances between panels in the door configuration.

## 5. Conclusions

To our knowledge, the MIC system represents a breakthrough in the world of MIMO radar imaging, as it is the first-of-its-kind low-cost system for imaging walking persons using COTS elements. The system boasts a transmission time of 70 ms and a total measurement time of approximately 165 ms, allowing for quasi-real-time measurements of walking persons.

The MIC system has been installed and actively employed in the DEXTER system prototype, a multi-sensor detection system, at the Anagnina metro station in Rome, Italy. The system has successfully performed discreet experiments by scanning over 550 people carrying different objects, achieving a success rate of 95% in these measurements.

The MIC system can provide 2-cm resolutions at a distance of 150 cm from the array aperture. The system’s power consumption is 72 W/h, and it illuminates using a power density significantly lower than the required power density level of 10 W/m² for members of the public.

The MIC system is currently operational, producing 2.8 frames per second of 3D radar images. Once the image reconstruction time (currently about 350 ms) is reduced in the future, the system will be able to produce 3D radar images at 6 fps. The reconstructed range is currently within 50 cm to 250 cm with 100 points. The reconstructed number of voxels in the z-axis can be reduced by considering the range position of a walking person to reduce the reconstruction time. 

Lastly, antenna configuration and discretion could be improved by enlarging the door width or moving to a fully discreet wall configuration. However, these tasks represent significant scientific challenges that will require further research.

## Figures and Tables

**Table 1:** System hardware parameters.

Parameters	
Module—Frequency Range	6.5 GHz–10.5 GHz
Module—Number of Frequencies	81 points
Total number of transmitter antennas	352
Total number of receiver antennas	528
Total number of modules	24

**Table 2:** MIC fail cases, occurrences appear in parentheses.

Confusions	False Positives	False Negatives
Small gun instead of big gun (2)	Small gun on woman (2)	Big gun on woman (2)
Explosive belt instead of a Backpack on the front (3)	Explosive belt on woman (1)	Small gun on woman (2)
AK47 instead of a big Metallic Bottle on the front (1)	AK47 on woman with arms crossed (1)	Small gun on man (3)
	Small gun on “phone texting” (1)	Explosive belt on woman (1)
	Big gun on “phone texting” (1)	Explosive belt on man (2)
	Umbrella confused with small gun (1)	AK47 in wrong position
